# Gastric Mucosal Lesions in Tibetans with High-Altitude Polycythemia Show Increased HIF-1A Expression and ROS Production

**DOI:** 10.1155/2019/6317015

**Published:** 2019-03-14

**Authors:** Kang Li, Chaohui He

**Affiliations:** ^1^High Altitude Medical Research Institute, People's Hospital of Tibet Autonomous, China; ^2^The Fifth Affiliated Hospital of Zunyi Medical University, Zhuhai 519100, China

## Abstract

**Background:**

Living at a high plateau in a very hostile environment and low oxygen levels often leads to the development of high-altitude polycythemia (HAPC) and gastric mucosal lesions caused by high-level reactive oxygen species (ROS). Hypoxia-inducible factor-1A (HIF-1A) helps maintain oxygen homeostasis by promoting the transcription of various genes and can be affected by ROS levels. To evaluate the molecular mechanism by which HAPC causes the gastric mucosal lesions, the expression of HIF-1A was measured in Tibetans with HAPC and in healthy subjects. Ultrastructural, histopathological, and immunohistochemical analyses were performed in the gastric tissues of both groups, and the expression of HIF-1A in the gastric mucosa was detected using qPCR and Western Blot.

**Results:**

The microvessel density and average diameter of gastric mucosal vessels were significantly greater in the HAPC patients than in the healthy subjects (*p* < 0.05). The number of red blood cells in the gastric mucosa was also significantly higher in the HAPC group than in the healthy subjects (*p* < 0.05). In addition, the density of the mitochondrial vacuoles and endoplasmic reticulum and pathological apoptosis were significantly increased in the gastric cells from HAPC patients compared to those from the healthy subjects. The levels of ROS and HIF-1A in the gastric mucosa were increased in HAPC patients compared to those in controls (*p* < 0.05).

**Conclusions:**

An increased level of HIF-1A was associated with HAPC development in the stomach of Tibetans living at a high altitude. ROS upregulated the levels of HIF-1A. Thus, ROS-mediated HIF-1A signaling transduction may be the mechanism associated with HAPC-induced gastric lesions.

## 1. Background

Living at a high plateau with a very hostile environment and low oxygen levels [1] can result in a series of physiological effects on the structure of gastric tissues, and physiological functions that are associated with high-plateau disease and major health problems may be impaired [2, 3]. The gastrointestinal tract is commonly affected and has greater sensitivity to hypoxia. Long-term and high-altitude hypoxia has varying effects on the digestive system [4].

Red blood cells play a central role in normal physiological processes in human tissues and organs by delivering oxygen and removing waste. The concentration of red blood cells should be maintained at a certain level [5]. Erythropoietin (EPO) is necessary for the growth and survival of erythroid progenitor cells and production of red blood cells. The EPO level is regulated in response to oxygen tension [6, 7].

Hypoxia-inducible factor-1 (HIF-1) is a transcription factor, consisting of *α* and *β* heterodimer subunits, and is coded by the* HIF-1A* gene. HIF-1A acts as the main regulator of homeostasis in hypoxic cells by activating the transcription of various genes, including those involved in energy metabolism [8, 9], angiogenesis [10, 11], and apoptosis [12, 13], as well as other genes involved in increasing oxygen delivery or promoting metabolism under hypoxic conditions [14, 15]. Therefore, HIF-1A plays an important role in the pathophysiology of ischemic diseases [16, 17]. HIF-1A is activated in hypoxia and promotes the transcriptional activation of downstream genes, including EPO [18, 19], leading to the increase in red blood cell production.

The mechanism underlying hypoxia-induced morphological changes in the microstructure of gastric tissues and the role of HIF-1A in the pathogenesis of high-altitude polycythemia (HAPC) in Tibetans remain unclear. Therefore, we investigated the effects of a high-altitude hypoxic environment on physiological function in human gastrointestinal mucosa and evaluated the molecular mechanisms associated with HIF-1A in the pathogenesis of HAPC.

## 2. Methods

### 2.1. Participants

All protocols were approved by the Human Research Ethics Committee of People's Hospital of Tibet Autonomous Region (Approval No. 20150108x2, Lhasa City, People's Republic of China). Written informed consent was obtained from all participants. The studies were performed according to the Guidelines of the Declaration of Helsinki and Ethical Principles for Medical Research Involving Human Subjects.

The severity of mucosal lesions was classified according to HE staining of gastric tissues and red blood cell aggregation: (1) controls had healthy gastric tissues without red blood cell aggregation or leakage, and or seepage in gastric tissues; (2) HAPC patients had mucosal lesions with oozing blood red cell clustering (clusters of > 3 erythrocytes). The categories were determined according to the number of red blood cells and vessels, degree of red blood cell aggregation, and the pathological findings. In July 2013, 24 HAPC patients who lived on the Tibetan Plateau at an altitude of 3600 m to 4800 m, were assigned to the HAPC patient group. Complications in the HAPC patients were assessed with gastroscopy. In addition, 21 healthy Tibetans from the same altitudes were assigned to the control group. All subjects were from Lhasa, Nagqu, Shannan, or Rigaze in Tibet. All subjects were indigenous and had lived in the same locations for more than 30 years. Every subject was matched with a control based on sex, place of residence, age, body mass index (BMI), and life habits among others. As BMI and daily caloric intake are major contributors to gastric injury [20], so these components were carefully measured. All patients were male native Tibetans from Lhasa with age ranging from 40 to 45 years. All patients provided written informed consent before tissue sampling.

Peripheral venous blood was obtained and oxygen saturation was detected using pulse oximetry. Inclusion criteria for the study were HAPC diagnosed according to the definition given by 2004 Qinghai International High Altitude Medicine Conference, including hemoglobin (Hb) concentration > 21 g/dL (males) and > 19 g/dL (females) [21]. Exclusion criteria were chronic pulmonary disorders, such as emphysema, bronchitis, bronchiectasis, alveolar fibrosis, lung cancer, and other serious pulmonary diseases; serious respiratory diseases or secondary polycythemia due to hypoxemia associated with documented chronic disease; intestinal tract obstruction; and history of intestinal bleeding. The diagnosis of chronic gastritis was based on the guidelines developed by the Chinese consensus on chronic gastritis [22]. Histopathological diagnosis was performed according to the gastritis assessment staging system [23].

### 2.2. Endoscopic Observation

The upper gastrointestinal tracts of all participants were observed using endoscopy [24]. HAPC was examined after ingestion of 10-mL 2% lidocaine HCl viscous solution (Jiangsu Jichuan Pharmaceutical Co., Ltd., China). All subjects were observed under a gastroscope (OLYMPUS GIF-260, Olympus, Japan) by a professional endoscopist. The same light source and endoscopy lamps were used during the whole experiment. The color of the upper gastrointestinal tracts was observed under an endoscope and color changes were photographed. Meanwhile, mucosal biopsy was taken from all participants.

### 2.3. Histological Analysis

Mucosal sampling was performed in the Digestive Endoscopy Department, People's Hospital of the Tibet Autonomous Region (Tibet, China). We analyzed 45 gastric antrum biopsy specimens, of which 24 were from HAPC patients and 21 from controls with normal mucosa. The gastric mucosal samples obtained with forceps biopsy were flash-frozen in liquid nitrogen immediately and stored at -80°C until use. Gastric mucosal tissues were fixed in formalin and embedded in paraffin for histological analyses. Five-*μ*m sections were stained with H&E (Bi Yuntian, China). Blood vessels and red blood cells in microvessels were assessed with a 400× magnification, and the diameter of gastric mucosal vessels was measured. The preserved samples were prefixed in 3% glutaraldehyde, followed by 1% osmium tetroxide fixation, gradual dehydration in gradient acetone, Epon812 embedding, optical positioning of semithin sections, slicing of ultrathin sections, and double staining with both uranyl acetate and citrate lead. Finally, the sections were photographed with a transmission electron microscope (Hitachi H-600IV, Japan). The results were evaluated by 2 researchers in a blinded fashion. The mucosa was considered destroyed if one of the following was apparent: discontinuous surface, dilated glands, hemorrhage, or superficial cell damage [25].

### 2.4. Immunohistochemical Analysis

Gastric mucosal cells were cultured in hydrogen peroxide solution for 15 min, and 100 *μ*L of normal serum was added, with further culturing for 10 min. Anti-HIF-1A antibody (rabbit anti-human, 1:200 dilution, ProteintechTM, ABCAM, USA) was added, and the cells were incubated overnight (16 h) at 4°C. The mixture was washed 3 times with PBS. Horseradish peroxidase goat anti-rabbit secondary antibody (goat anti-rabbit, 1: 200, ProteintechTM, ZSGB-BIO, China) was added and incubated for 30 min. Finally, the mixture was washed 3 times. The staining was developed with a DAB color kit (ZSGB-BIO, China). Astrocytes were stained with hematoxylin and whole-mounted. The expression and distribution of HIF-1A were observed in the tissue.

### 2.5. RNA Extraction

Gastric mucosal samples from 24 HAPC patients and 21 controls were pooled for each genotype. Each pool was placed in 3-mL of Trizol (Shanghai Sangon Co., China) and grounded with a glass homogenizer. After extraction with chloroform, RNA was precipitated with isopropanol. The final mixtures were placed into TE buffer (Tris-HCl buffer, pH 8.0, containing 1.0 mM EDTA) and digested with DNase; RNA quality was evaluated at 260/280 nm using a spectrophotometer (NanoDrop 1000, NanoDrop Technologies, Wilmington, DE, USA).

### 2.6. Real-Time qRT-PCR

Chip results were reevaluated using real-time qRT-PCR for the differentially expressed* HIF-1A* gene, with the *β*-actin gene as a control. Total RNA from gastric mucosal samples was purified with a Trizol reagent kit (Life Technologies, Maryland, CA, USA). Five-*μ*g of RNA was reverse-transcribed with the One-Step PrimeScript cDNA Synthesis Kit (Takara, Dalian, China). Platinum TaqDNA polymerase was purchased from ABI (USA). The primers were designed as follows: HIF-lA, forward primer, 5′-ACCCTCTGATTTAGCATGTAG-3′, reverse primer, 5′-GTAGGTTTCTGCTGCCTTGT-3′; Probe, 5′-FAM- CTGGTCAGCTGTGGTAATCC-TAMRA-3′; *β*-actin, forward primer, 5′- GAAGATCAAGATCATTGCTCCT-3′, reverse primer, 5′- TACTCCTGCTTGCTGATCCA-3′; probe, 5′-FAM-CTGTCCACCTTCCAGCAGA-TAMRA-3′; PCR was repeated three times using SYBR Green PCR Master Mix and the reactions were performed in 7500 Fast real-time PCR detection system (ABI Biosystems, Foster City CA, USA) under the following amplification conditions: 94°C for 2 min, 45 cycles at 94°C for 20 s, 53°C for 30 s, and 60°C for 45 s. Relative expression value was calculated with the 2^-∆∆T^ method.

### 2.7. Western Blot Analysis of HIF-1A

Glandular epithelial cells of gastric mucosa were extracted and the protein levels of HIF-1A were analyzed using Western Blot. The total protein was obtained using cocktail after the cells were lysed for 3 times by freezing and thawing [26]. Supernatant protein was separated using SDS-PAGE and transferred to a PVDF membrane, and blocked by 2.5% nonfat dry milk for half an hour. The membrane was subsequently incubated with HIF-1A antibodies (diluted 1:1000) overnight at 4°C. An ABC peroxidase and peroxidase substrate kits were used to examine the primary antibodies. Using X-ray film exposure and photographs, expression of HIF-1A was measured using Quantity One with *β*-actin as an internal control.

### 2.8. Terminal Deoxynucleotidyl Transferase-Mediated Deoxyuridine Triphosphate Nick-End Labelling (TUNEL) Analysis for Apoptosis

The slides of mucosal issues were treated with 10 *μ*g/ml proteinase K (Sigma, St Louis, MO, USA) for 15 min at RT and washed for 2 min with ddH_2_O. Endogenous peroxidases were deactivated by 2% of hydrogen peroxide for 5 min. The sections were rinsed with ddH_2_O, and immersed in TDT buffer [26]. TDT (200 U/mL; Takara, Tokyo, TUNEL assay for apoptosis of gastric mucosa) and biotinylated dUTP (0.01 mM BMY) in TDT buffer were then added to the sections, and incubated in a humid atmosphere at 37°C for 1 h. The reaction was stopped by using TB buffer (0.3 M NaCl, 0.04 M sodium citrate) for 20 min. The sections were rinsed with ddH_2_O, covered with 10% BSA for 10 min, then rinsed in ddH_2_O, and immersed in PBS for 5 min. The sections were incubated with horseradish peroxidase (HRP) (Sigma) for 50 min, then washed with 20 *μ*M PBS and 150 mM NaCl, and treated with 0.02% DAB. After being rinsed in ddH_2_O, the slides were stained with hematoxylin for half an hour at 37°C. Dehydration was performed according to a previous report [26]. The tissue sections were then mounted in Permount (Fisher Scientific, Pittsburgh, PA, USA). Apoptotic cells identified by the TUNEL assay were quantitated under brightfield microscopy by a single observer. Cell number was counted under high power using 6 different views from each specimen. The POD apoptosis detection kit (Roche, USA) was used in accordance with instructions. Paraffin sections of gastric mucosa were TUNEL stained and maximized at 400× under a microscope.

### 2.9. Assay of Intracellular ROS

Intracellular ROS levels were determined using the dichloro-dihydro-fluorescein diacetate (DCFH-DA) method according to a previous study [27]. Isolated gastric mucosal cells were incubated with DCFH-DA in PBS buffer at 37°C for 30 min. Subsequently, the buffer was removed and the samples were washed 3 times using the same buffer. The fluorescence absorbing values were measured by using a fluorescence plate reader. Excitation wavelength was 485 nm and emission wavelength was 535 nm. Relative ROS values were compared between the patients and healthy subjects.

### 2.10. Statistical Analysis

All results were presented as mean ± SD. SPSS11.5 statistical software was used for analysis of variance. A chi-square test was used for number analysis and a Student's t-test was used for normally distributed data analysis after testing for normality by means of Kolmogorov Smirnov test in the present study. One-way analysis of variance (ANOVA) was used to compare variables between two groups. The post hoc test was the Newman-Keuls test. Sample power was calculated by using STATA 10 (StataCorp, College Station, TX, USA). The level of significance is set a priori if *p* < 0.05.

## 3. Results

### 3.1. Baseline Participant Characteristics

Baseline and physical characteristics of the study population were shown in Tables 1 and 2. The statistical difference was insignificant for living altitude, age, smoking, drinking, BMI, or duration at different altitudes between HAPC subjects and controls (*p* > 0.05, Table 1). Hb, EPO, ROS and diastolic pressures in the HAPC group were higher than in the control group (*p* < 0.01). In contrast, oxygen saturation was lower than in a control group (*p* < 0.01). No significant difference in other parameters was found between the control and experimental groups (Table 2).

### 3.2. Endoscopic Upper Gastrointestinal Tract Examination

**Figure 1:**
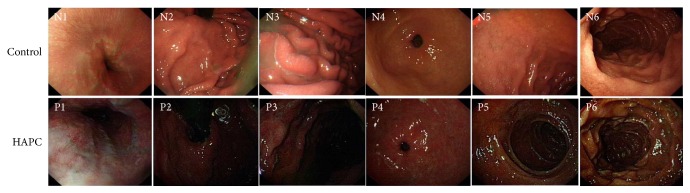
Endoscopic findings of an upper gastrointestinal (upper GI) series. N1/P1, esophagus; N2/P2, cardia; N3/P3, gastric fundus; N4/P4, antrum stomach body; N5/P5, duodenal bulb; and N6/P6, descending portion. N stands for healthy subjects and P presents for polycythemia ones.

The HAPC group was found to have mucosal hyperemia and edema with a darker color in the upper gastrointestinal tract, including the esophagus (N1/P1), gastric fundus (N2/P2), gastric body (N3/P3), gastric antrum (N4/P4), duodenal bulb (N5/P5), and descending duodenum (N6/P6) (87.5% versus 4.8%, p<0.001) (Figure 1). Moreover, in the HAPC group, the esophageal mucosa became thinner and redder and fine vascular nets could be found under the mucosa as cord-like and branchlike structures. Furthermore, the veins became wider in the observed area. In addition, deepened pink of the esophageal mucosa destroyed the boundary that could be distinguished between esophageal and stomach mucosa.

**Figure 2:**
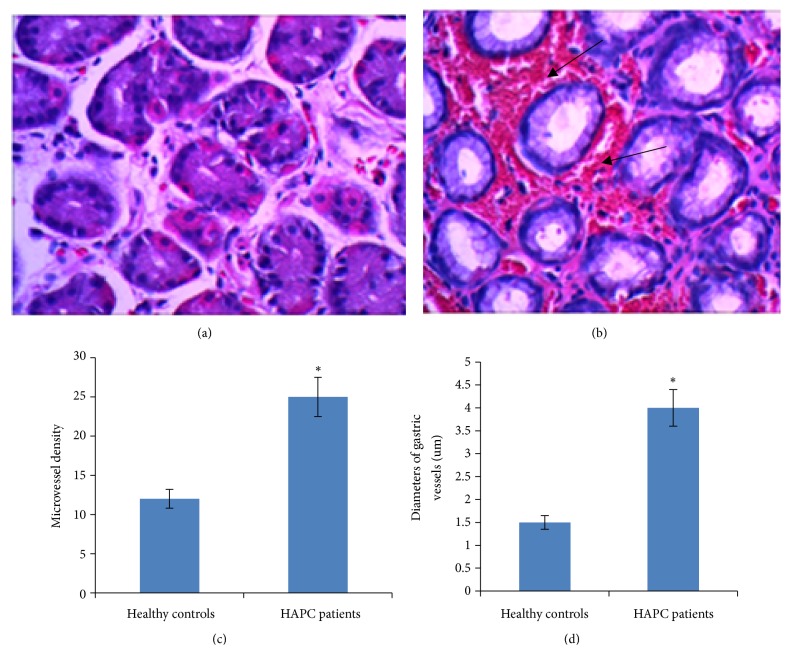
HE staining comparison for gastric biopsy between HAPC patients and healthy controls. (a) For the gastric biopsy from healthy controls, the duct is in normal structure with suitable amounts of blood vessels and red blood cells; (b) for the gastric biopsy from HAPC patients, the duct is destroyed. A large number of red blood cells were gathered in gastric vessels. Parietal cells are overproliferated in gastric biopsies. The number of gastric cells is decreased while the diameter of gastric mucosal vessels is increased compared with healthy controls. (c) Microvessel density (MVD). (d) Average diameter of gastric mucosal vessels. *∗p *< 0.05 via controls.

In the HAPC group, gastric mucosa was dark red, or red-purple, and diffuse hyperemia and edema as well as significant changes of congestion. In the duodenal bulb and descending parts of upper gastrointestinal tract in the HAPC group, the mucosa was browner and brownish red when comparing with controls. In addition, the villi in the duodenal bulb became enlarged with hyperemia and swelling (Figure 2).

### 3.3. H&E Staining of Gastric Biopsy Specimens from HAPC Patients and Healthy Controls

In gastric biopsy specimens from the controls, the ducts appeared normal with appropriate numbers of blood vessels and red blood cells (Figure 2(a)). In gastric biopsy specimens from HAPC patients, the ducts were destroyed. A large amount of red blood cells was aggregated in gastric vessels. Parietal cells showed overproliferation in gastric biopsies. The amounts of gastric cells were reduced while the diameters of gastric mucosal vessels were increased when comparing with the controls (Figures 2(a) and 2(b)).

### 3.4. Number of Gastric Vessels

Statistical analysis showed that gastric microvessel density (MVD) was significantly greater in HAPC patients than in healthy controls (24.33 ± 4.58 and 12.00 ± 3.03) (Table 3). There were significant differences between the two groups (P <0.05) (Figure 2(c)).

### 3.5. Average Diameter of Gastric Mucosal Vessels

Statistical analysis showed that the average diameter of gastric mucosal vessels in the stomach of HAPC patients was significantly greater than in healthy controls (3.90 ± 1.31 and 1.63 ± 0.56). There were significant differences between the two groups (*p* < 0.05) (Figure 2(d), Table 3).

### 3.6. Number of Erythrocytes in Gastric Mucosa

Statistical analysis showed that the number of erythrocytes in gastric mucosa of HAPC patients was significantly higher than in healthy controls (149.58 ± 69.63 and 29.95 ± 16.79). There was a significant difference between the two groups (*p* < 0.05) (Table 3). The erythrocytes were not only distributed in blood vessels, but also in tissue spaces, suggesting that high altitude increases the number of gastric vessels and causes stomach injury.

### 3.7. Microstructure of Gastric Mucosa

**Figure 3:**
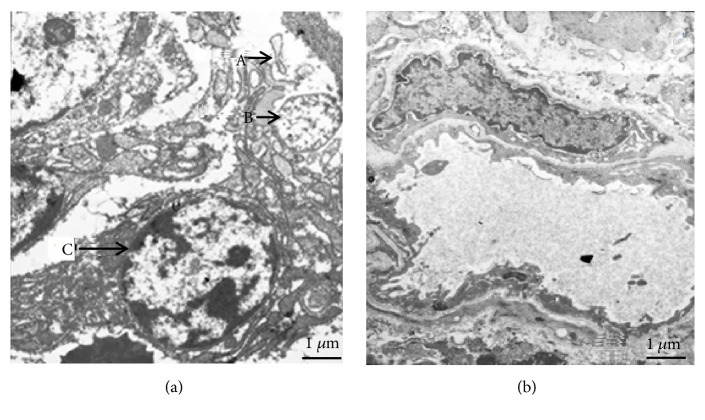
Ultrastructure of the gastric mucosa in the high-altitude polycythemia group double staining of both uranyl acetate and lead citrate ×10 000. Transmission electron microscopy of epithelial cells of the gastric mucosa in high-altitude polycythemia group (a), swelling of endoplasmic reticulum (A), mitochondria edema (B), and lymphocytic infiltration (C); control group (b), the epithelial cell structure of the gastric mucosa appeared normal.

Transmission electron microscopy (TEM) showed normal gastric microstructure in healthy controls while significant changes were observed in HAPC patients. The endoplasmic reticulum was increased while mitochondria became swollen and degenerated vacuoles were found in HAPC patients (Figure 3). TEM revealed various degrees of expansion of the endoplasmic reticulum, as well as mitochondrial swelling in epithelial cells of the gastric mucosa in the HAPC group. In addition, some epithelial cells disappeared and demonstrated development of vascular degeneration. Some of the cell nuclei were damaged and lymphocytic infiltration was observed. In contrast, in the control group, the epithelial cell structure of the gastric mucosa appeared normal (90.9% versus 8.3%,* p* < 0.001) (Figure 3).

### 3.8. Immunohistochemical Analysis of HIF-1A

**Figure 4:**
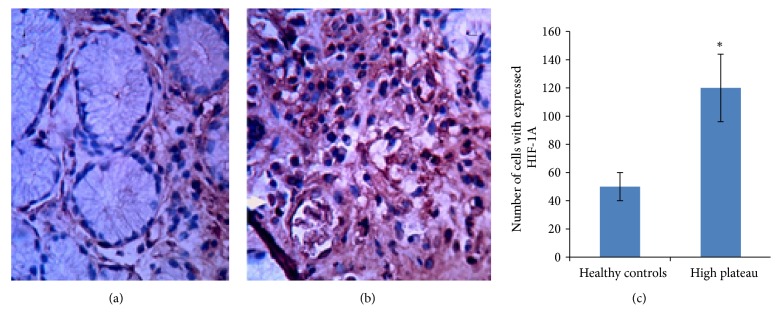
Immunohistochemistrial analysis for the expression of HIF-1A. (a) Normal gastric mucosa from healthy controls. (b) Gastric mucosa from HAPC patients. (c) *∗*P <0.05 via controls.

Immunohistochemistry showed that the levels of HIF-1A in gastric mucosa of HAPC patients were significantly higher than those from healthy controls. There were significant differences between the two groups (*p* < 0.05) (Figure 4).

### 3.9. The Effects of HAPC on the mRNA Levels of HIF-1A

**Figure 5:**
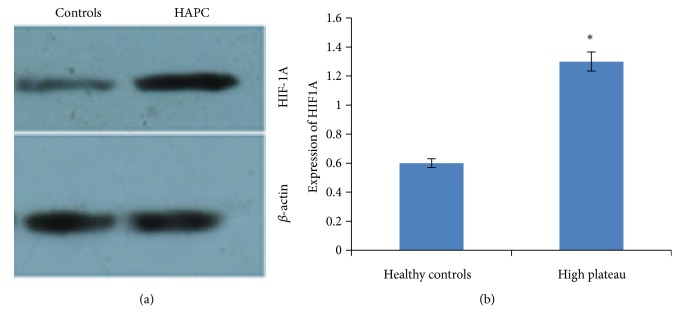
The expression of HIF-1A. (a) qPCR analysis for the mRNA levels of HIF-1A. *∗*P <0.05 via controls. (b) Western Blot analysis of the protein levels of HIF-1A. (a) The comparison for the protein levels of HIF-1A between HAPC patients and healthy controls. (b) *∗p* <0.05 via controls.

qPCR analysis showed that the mRNA levels of HIF-1A in gastric mucosa of HAPC patients were significantly higher than in healthy controls. There were significant differences between the two groups (*p* < 0.05) (Figure 5(a)).

### 3.10. Effects of HAPC on the Protein Levels of HIF-1A

Western Blot analysis showed that the protein levels of HIF-1A in gastric mucosa of HAPC patients were significantly higher than in healthy controls. There were significant differences between the two groups (*p* < 0.05) (Figure 5(b)).

### 3.11. TUNEL Analysis

**Figure 6:**
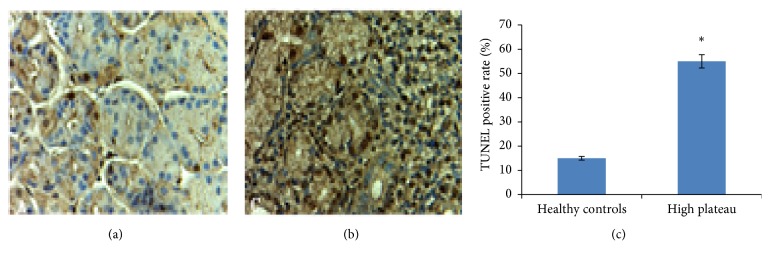
TUNEL analysis of levels of HIF-1A in gastric mucosa. (a) The levels of HIF-1A in gastric mucosa from healthy controls. (b) The levels of HIF-1A in gastric mucosa from HAPC patients. (c) The comparison for apoptotic gastric cells between HAPC patients and healthy controls. *∗p* <0.05 via controls.

TUNEL analysis showed that apoptosis in gastric mucosa was significantly greater in HAPC patients than in healthy controls. There were significant differences between the two groups (*p* < 0.05) (Figures 6(a) and 5(b)).

### 3.12. Apoptotic Analysis

Analysis showed that the levels of apoptotic gastric cells in the stomach of HAPC patients were significantly greater than in healthy controls. There were significant differences between the two groups (*p* < 0.05) (Figure 6(c)).

### 3.13. Intercellular ROS Concentration

**Figure 7:**
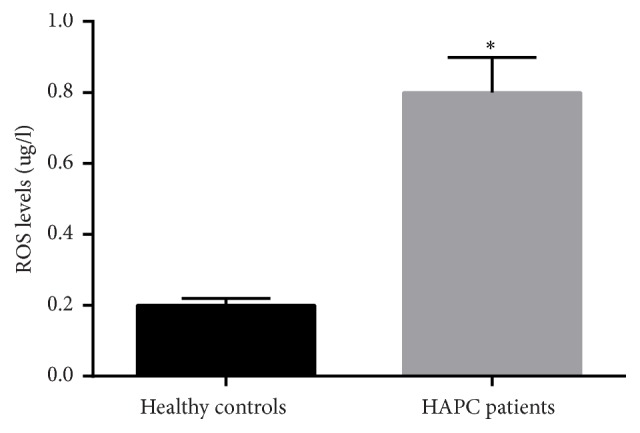
ROS generation in different cells from between HAPC patients and healthy controls. All the data were represented as the mean values ± SD. *∗p* < 0.05 via a healthy subject.

ROS levels were significantly higher (to 220%) in the cells from healthy subjects (*p *< 0.05, Figure 7). The results were consistent with previous reports: ROS are important risk factors for polycythemia at high altitude.

## 4. Discussion

Classic high-altitude physiological responses include hyperventilation [28] and polycythemia [29, 30]. The gastrointestinal tract is more sensitive to hypoxia [31]. Thus, hypoxia can have varying degrees of influence on the gastric mucosa and polycythemia is the main pathology of HAPC-induced disorders in most Tibetans [30, 32]. Our results showed a significant increase in the number of red blood cells, gastric vessels, and diameters of gastric mucosal vessels in HAPC patients when compared with healthy controls. Furthermore, more red blood cells were distributed in gastric tissue not only at vascular levels but also in tissue spaces. The number of vacuoles was increased in gastric mucosal cells. Meanwhile, the number of mitochondria and the endoplasmic reticulum were also increased. Moreover, there was a significant increase in apoptosis of gastric mucosal cells. These pathologic changes show the effect of high-altitude hypoxia on the human gastrointestinal mucosal barrier, leading to adverse effects on physiological, and immune function, among others.

Comparing with our reported work [26, 33], the population size was increased from 6 and 24 to 45 subjects in the present study. Furthermore, the color of brown lesions under the mucosa in upper gastrointestinal tract was much darker than before reported in HAPC patients (Figure 1). Present findings showed that there were 21 cases (87.5%) and 1 case (4.8%) with darker brown color under the gastrointestinal mucosa in the HAPC and control groups (p<0.001), respectively. The color-based biomarker detection can be used for the diagnosis of HAPC but the data could not be obtained from previous work with smaller population.

Hypoxia induces HIF-mediated gene expression [34]. Hypoxia promotes caspase-3 activation [35] and will increase the apoptosis of gastric mucosal cells. Hypoxic states can be manipulated if oxygen is increased [36]. The increase in partial pressure of oxygen (ppO_2_) and normobaric air [37] can suppress the expression of caspase-3 [38] and will inhibit the apoptosis of gastric mucosal cells.

Production of HIF is the body's response to hypoxia and important in adaptation [39–42]. HIF production has an obvious impact on the expression of other genes. qPCR, Western Blot and immunohistochemical analyses showed that the levels of HIF-1A in the gastric mucosa of HAPC patients were significantly higher than in healthy controls. There were significant differences between the two groups (*p *< 0.05) (Figures 5 and 6).

**Figure 8:**
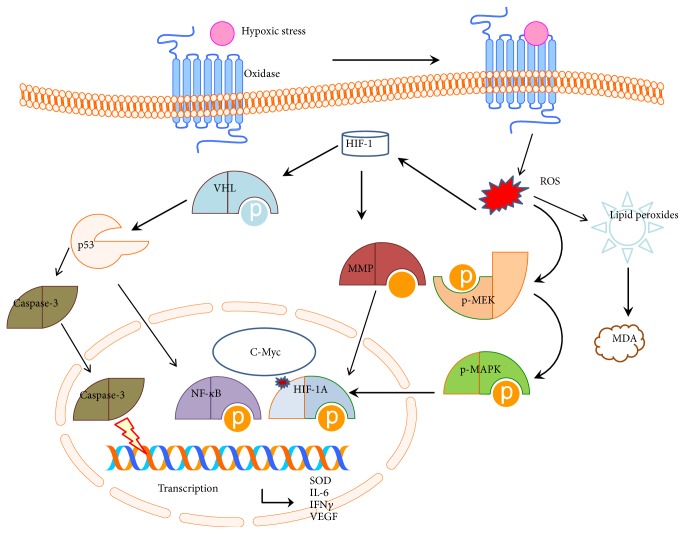
The schematic diagram of ROS-medicate HIF-1 signaling pathway.

High attitude hypoxic conditions will significantly increase ROS [43, 44] (Figure 8). The increased ROS act as signal transduction messengers that induce stabilization of HIF-1A, which is important in maintaining normal physiologic responses under hypoxic conditions [45]. HAPC is closely associated with angiogenesis [46], which will affect the development of gastric mucosal lesions. The c-Myc/HIF-1A signaling axis (Figure 8) can reduce lesion-stimulated angiogenesis [47]. An association between Von Hippel Lindau (VHL) gene and the HIF pathway has been reported in some human disorders [48]. HIF-1 inhibits mitochondrial biogenesis and O^2^ consumption in cells lacking the VHL gene by inhibiting c-Myc activity [49] (Figure 8). The loss of VHL protein will result in impaired p14/ARF function and suppression of p53 expression [50]. p53 and caspase-3 are the main biomarkers in apoptotic-signaling pathways [51] and can be affected by HIF-1A (Figure 8). All these molecules will affect the expression of SOD, IL-6, IFN and VEGF and result in changes of antioxidant, anti-inflammatory and antiapoptosis in gastric cells (Figure 8). HIF-1A can cause ischemia-reperfusion-induced gut mucosal injury. HIF-1A responses in gastric mucosa may be potentially maladaptive or damage gastric barrier function and result in other organ injury according to previous report [52]. All results have suggested that high-level expression of HIF may lead to HAPC-induced gastric mucosa lesion and is an important ROS-mediated signaling pathway.

There were some limitations in the present work. No experiment was performed to determine whether ROS actually exacerbated gastric mucosal lesions or to determine a role for HIF-1A. Our work showed an increase of both in the gastric mucosa but not the role they play. Further work is needed to address this issue.

## 5. Conclusions

In sum, MVD, the diameters of gastric mucosal vessels, and the number of apoptotic cells were significantly greater in HAPC patients than in healthy controls. This study also suggested a molecular mechanism for the pathogenesis of HAPC. The levels of HIF-1A in gastric mucosa of HAPC patients were significantly higher than in healthy controls (*p* < 0.05). The results provide a basis for pathogenesis of gastrointestinal mucosal lesions in HAPC patients.

## Figures and Tables

**Table 1:** The baseline characters of participants.

Characteristic	Control	HAPC	*t*-value	*p*-Value
Age (years)	42.5 ± 2.5	42.3 ± 2.3	0.3645	0.3819
Smoking (no/yes)	4/10	3/8	0.1254	0.2465
Drinking (no/yes)	5/9	4/7	0.2487	0.4526
BMI	32.4± 3.5	33.5 ± 2.8	0.2241	0.3708
Living at altitude (meters)	4557 ± 668	4523 ± 623	0.8509 21.	0.2168
Time on high plateau (years)	34.4 ± 4.2	35.6 ± 5.1	0.2324	0.3682

Note: BMI, body mass index. There is a significant difference if *p* < 0.05.

**Table 2:** Basic physiological indices in participants.

Characteristic	Control	HAPC	*t*-value	*p*-Value
Hb (g/dl)	21.54 ± 2.34	22.85 ± 2.17	2.3452	0.0126
EPO (ng/ml)	1.53 ± 0.12	1.78 ± 0.14	5.6541	0.0168
ROS (fluorescence intensity)	521.4 ± 20.6	651.2 ± 30.9	5.8801	0.0012
Systolic pressure (mmHg)	112.4 ± 11.6	121.1 ± 14.5	2.9083	0.0748
Diastolic pressure (mmHg)	74.2 ± 8.8	88.2 ± 10.1	4.1258	0.0225
Blood oxygen saturation (%)	90.2 ± 2.3	84.1 ± 2.8	5.4618	0.0064
Heart rate (time/mini)	78.1 ± 4.2	79.6 ± 5.1	2.0786	0.1342

Note: there is a significant difference if *p* < 0.05.

**Table 3:** Characters of gastric mucosal vessels.

		Number of vessels		Average diameter of vessels		The number of erythrocyte (400×)	
Groups	Slices	Average	SD	Average	SD	28.71	11.71
Healthy controls	21	12.16	2.64	11.73	2.80	154.63	46.24
HAPC patients	24	25.78	3.60	33.60	9.80	28.71	11.71

## Data Availability

The data used to support the findings of this study are available from the corresponding author upon request.
